# Unexplained Chronic Liver Disease and Hemolytic Anemia in a Young Girl: A Case of Wilson’s Disease

**DOI:** 10.7759/cureus.50724

**Published:** 2023-12-18

**Authors:** Abdul Wahab, Kriti Sapkota, Karthik Jayakumar, Ebad-Ur Rehman Syed, Rooh Ul Amin, Hidayat Ullah, Nauman Khan

**Affiliations:** 1 Internal Medicine, Hayatabad Medical Complex Peshawar, Peshawar, PAK; 2 Internal Medicine, Medical University of Gdańsk, Gdańsk, POL; 3 Surgery, Medical University of Gdańsk, Gdańsk, POL; 4 Medical School, Royal College of Surgeons in Ireland, Busaiteen, BHR; 5 Medical Unit, Hayatabad Medical Complex Peshawar, Peshawar, PAK; 6 Internal Medicine, Khyber Medical College, Peshawar, PAK

**Keywords:** penicillamine therapy, hemolytic anemia, bicytopenia, ascites, wilson’s disease

## Abstract

Wilson's disease (WD) is an autosomal recessive disorder affecting the metabolism of copper that can present with a variety of clinical symptoms. Low levels of serum copper and ceruloplasmin, increased excretion of copper in the urine, and/or increasing quantities of copper in the liver are diagnostic indicators. The gold standard for diagnosis is genetic testing. The care approach includes the utilization of liver transplants as a therapeutic option in advanced patients and the use of copper-chelating medications. We describe a unique case of WD in a 14-year-old girl who presented with ascites, hemolytic anemia, and liver dysfunction. There was no indication of abdominal TB, and her viral, autoimmune, and hemolytic profiles were all normal. Low serum ceruloplasmin, elevated urine copper, and distinctive liver histology all supported the WD diagnosis. After starting penicillamine medication, the patient's symptoms improved, but her blood counts did not. This example emphasizes how crucial it is to rule out WD in patients with chronic liver disease, hemolytic anemia, and unexplained ascites, particularly in younger age groups.

## Introduction

Copper builds up in the body as a result of Wilson's disease (WD), a rare hereditary condition that mostly affects the nervous system, liver, and other critical organs [1]. A mutation in the ATP7B gene, which encodes for a protein that carries copper into the bile, results in WD. Because the disorder is autosomal recessive, a mutation in both of the two copies of the gene is required for the illness to develop [2,3].

In the majority of the time, WD signs and symptoms start in adolescence and typically occur between the ages of six and 45 [1,4]. Since the liver and brain are the primary locations where copper accumulates, liver illness and symptoms of neuropsychiatric disorders are the primary diagnostic criteria [2,4]. Compared to those with neuropsychiatric and mental symptoms, who often seek care in their 20s or later, people with liver disorders typically seek care sooner, typically as adolescents or teenagers [4].

Fatigue, jaundice, an increased propensity to hemorrhage, disorientation, features suggestive of portal hypertension, varices of the esophagus, splenomegaly, and ascites are some of the symptoms that liver disease might manifest as [1,4]. Tremors, muscular rigidity, difficulty speaking, changes in personality, anxiety, and psychosis are a few examples of neurological along psychiatric symptoms [1,4]. Hemolytic anemia and Kayser-Fleischer rings, a type of golden-brown eye discolorations brought on by copper deposits in the cornea, are additional signs of WD [1,2,4].

A mix of blood, urine, and liver biopsy tests are frequently used in the diagnosis of WD, which can be challenging [1,5]. Family relatives of individuals afflicted may be screened by genetic testing [1]. Low serum ceruloplasmin, elevated urine copper, and distinctive liver histology all support the diagnosis of WD [2,3,5]. Usually, medication and dietary modifications are used to treat WD. Dietary adjustments include avoiding copper cookware and adopting a low-copper diet. Zinc supplements and chelating agents, such as trientine and d-penicillamine, are among the medications utilized [6]. Neurological deterioration, renal issues, and liver failure are among the complications associated with WD. If alternative therapies are unsuccessful or liver failure develops, a liver transplant may be beneficial [5,6].

## Case presentation

A 14-year-old girl came into our outpatient clinic with the main complaints of discomfort, distention in her abdomen, and significant exhaustion during the previous two months. She also started to have breathing difficulties at the same time. She was pale when examined, and an examination of her abdomen revealed hepatosplenomegaly and shifting dullness to percussion.

Her family history and medical records were generally unremarkable, and there was no indication that she had ever used recreational drugs or over-the-counter health supplements. In addition, there was no family history of liver illness. Initially, the patient saw several doctors for ascites, but the majority of their investigations produced normal findings.

First laboratory tests revealed thrombocytopenia and hemolytic anemia, as Table 1 illustrates. Furthermore, elevated inflammatory markers were observed. A viral profile did not return any results. Examining the kidney function, heart, gastrointestinal tract, diet, and other organs implicated in the production of ascites were all part of the ascites workup, and all showed normal function. A mildly low albumin level, a slightly abnormal prothrombin time (PT), and an activated partial thromboplastin time (aPTT) were among the mild abnormalities revealed by liver function tests. The liver parenchyma on ultrasound showed coarse nodules with serrated borders, which is a sign of chronic liver disease. Vascular structures looked normal, and no focal mass lesions were seen.

**Table 1:** Baseline investigations Abbreviations: aPTT, activated partial thromboplastin time; ALP, alkaline phosphatase; Ast, aspartate aminotransferase; Creat, creatinine; Hb, hemoglobin; Na, sodium; MCV, mean cell volume; K, potassium; RBS, random blood sugar; WBC, white blood cells; LDL, low-density lipoprotein; BUN, blood urea nitrogen; TAG; triacyl glycerol, LDH, lactate dehydrogenase; PT, prothrombin time; Alt, alanine aminotransferase

Test	Patients value	Reference value
PT	15	<12
aPTT	29	<28
INR	1.2	1
Wbcs (/µl)	9500	4000 - 11000
MCV (fL)	85.5	80-100
Hb (g/dl)	9.3	11.5 - 17.5
LDH (U/L)	560	135 – 225
Platelets (/µl)	95000	150000 - 450000
Ferritin (ng/mL)	134	12 - 150
Transferrin saturation (%)	50%	20 -50 %
Alt (U/L)	67	10 - 50
Ast (U/L)	49	8 - 33
ALP (U/L)	377	< 390
BUN (mg/dl)	55	18 - 45
Bilirubin (mg/dl)	1.1	0.1 - 1
Creat (mg/dl)	0.9	0.3 - 0.9
Na (mmol/L)	134.9	135 - 150
K (mmol/L)	3.4	3.5 - 5.0
RBS (mg/dl)	110	100 - 125
LDL cholesterol (mg/dl)	66	<100
TAG (mg/dl)	114	<150
ESR (mm/hour)	56	0-20

Further testing was performed, including an autoimmune profile (anti-nuclear, anti-mitochondrial, anti-smooth muscle, and anti-parietal cell antibodies) to look into potential autoimmune reasons for liver failure and bicytopenia. Still, the outcomes were typical. Both the direct and indirect antiglobulin tests and the workup for further hemolytic anemia causes were normal.

**Figure 1:**
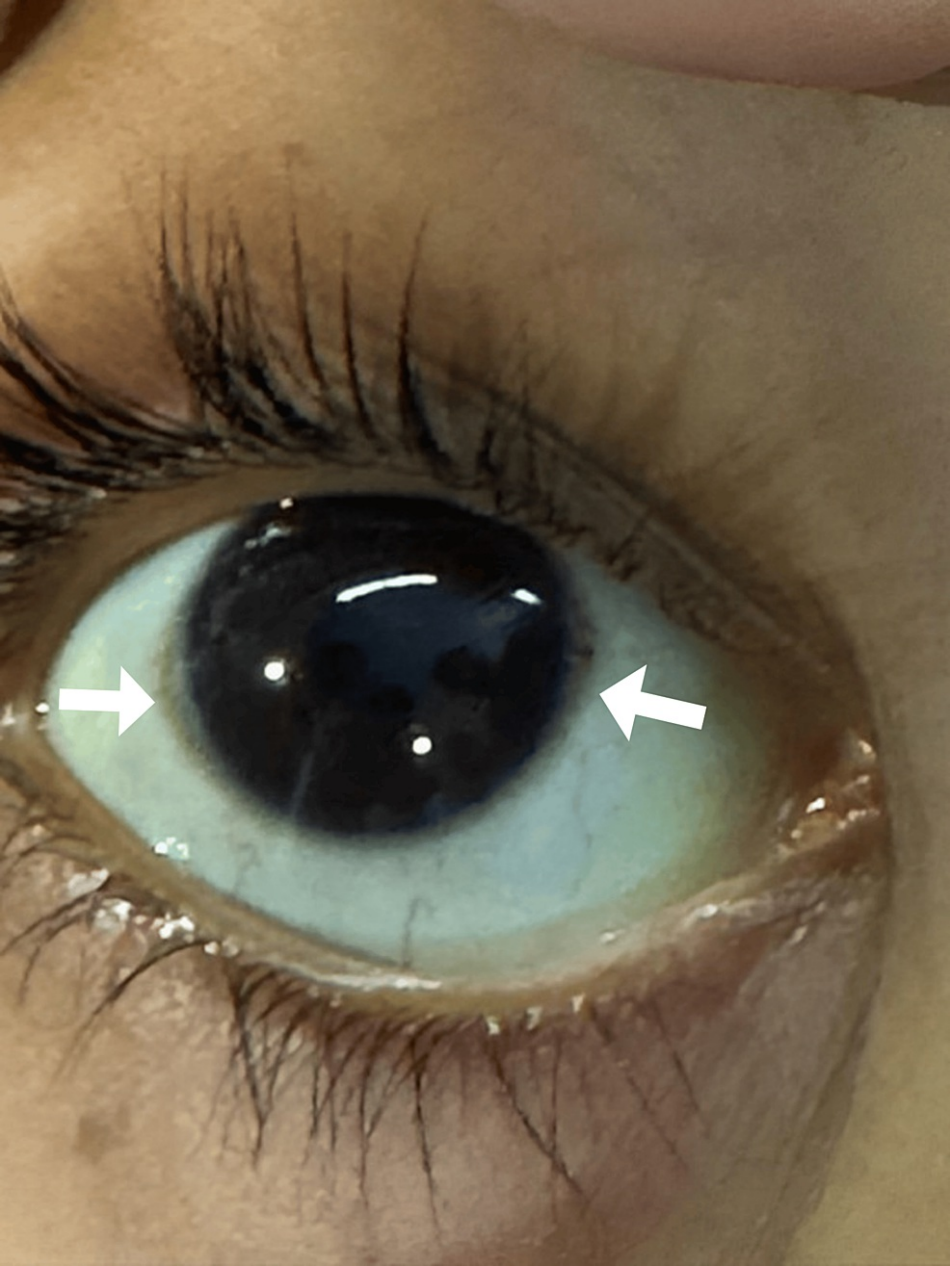
Kayser-Fleischer rings shown by white arrows

Table 2 shows that ascitic fluid routine inspection and serum ascites albumin gradient >1.1 excluded adenosine deaminase antibody (ADA) and atypical cells and implicated the liver as the origin of ascites. Abdominal tuberculosis was not found throughout the workup. A skepticism growing about WD prompted an ophthalmological examination of Kayser-Fleischer rings, as shown in Figure 1, in which brownish rings gave clues to WD. Along with it, serum ceruloplasmin was low and urine copper levels were high, which suggested WD as the underlying cause of bicytopenia and liver dysfunction.

**Table 2:** Specific laboratory investigations SAAG; serum ascites albumin gradient, ADA; adenosine deaminase

Investigation	Reference value	Patient value
SAAG ratio	>1.1 g/dL (portal hypertension) <1.1 g/dL (non portal hypertension)	1.4 g/dL
Ascitic fluid ADA levels	> 36 u/I	<5
24-hour urinary copper	<60	1595 µg/day
Serum ceruloplasmin	20-60	9 mg/dL

An examination of the liver confirmed WD by showing findings of anisonucleosis, ballooning change, and anisocytosis. With a Model for End-Stage Liver Disease-Sodium (MELD-Na) score of 15, the patient appeared to have a less than 2% chance of dying within the following 90 days. Penicillamine treatment was started, and the patient was advised to take medicine for the rest of her life. A follow-up appointment was set one month after this consultation. While the follow-up revealed an improvement in symptoms, blood counts remained abnormal, requiring further monitoring and modifications to the therapy regimen.

## Discussion

The diagnosis of WD is based on many clinical and biochemical evaluations. The classic presentation, which is characterized by identifiable Kayser-Fleischer rings and reduced serum ceruloplasmin levels, is often seen in people between the ages of five and 40 [7]. However, because 50% of those with liver problems do not match two of these three criteria, there are diagnostic hurdles that must be overcome [8].

A more methodical approach has been brought about by recent developments in diagnostics; recommendations to assist in the identification of liver diseases are provided by the American Association for the Study of Liver Diseases (AASLD). The patient exhibited symptoms that were in line with WD, such as ascites, hemolytic anemia, and early cirrhotic alterations. Prompted by clinical suspicion, further testing revealed normal renal function tests, inconclusive Kaiser-Fleischer rings, low ceruloplasmin (9 mg/dL), and increased 24-hour urine copper levels (1,595 µg/day). Remarkably, a sizable portion of WD patients have been shown to have urine copper levels <100 µg/day [9].

After a liver biopsy was carried out per protocols, anisonucleosis, ballooning change, and anisocytosis were discovered, supporting our suspicions. Unfortunately, genetic research was not carried out because of a lack of resources. Recent research highlights the intricacy of genetic variants by identifying more than 500 WD-causing mutations in the ATP7B gene. Remarkably, a single copy of a detectable point mutation may be present in as few as 25% of WD instances, highlighting the importance of alternate pathways for phenotypic expression [10,11]. Even though genetic testing is changing, it may still be used as a diagnostic tool, especially when there are unclear genetic results and additional laboratory results need to be taken into account.

In our situation, pathologic testing that focused on anisonucleosis was crucial. Histological evidence for WD was provided by the presence of glycogenated nuclei, ballooning change, anisonucleosis, and binucleation. This is similar to other research that found anisocytosis and anisonucleosis in cirrhotic livers on a regular basis [12]. Following AASLD recommendations, a thorough assessment of the patient's clinical presentation, test findings, and liver pathology was used to determine the diagnosis of WD [7].

## Conclusions

We have reported a rare case of WD in a 14-year-old girl who presented with hemolytic anemia, bicytopenia, and ascites. The patient did not have a family history of liver disease, nor had she ever used drugs or supplements. There was no indication of abdominal TB, and her viral, autoimmune, and hemolytic profiles were all normal. Low serum ceruloplasmin, elevated urine copper, and distinctive liver histology all supported the WD diagnosis. After beginning penicillamine medication, the patient's symptoms improved, but her blood counts did not. This instance emphasizes how crucial it is to rule out WD in patients with liver failure and unexplained ascites, particularly in younger age groups. If WD is identified early and treated properly, it can be cured. However, if ignored or misdiagnosed, it can result in life-threatening complications and even death. In order to stop irreparable damage, we should be knowledgeable about the clinical signs and diagnostic procedures associated with WD.
